# Optimization of forward pulsed currents for combining the precision shaping and polishing of nickel micro mould tools to reduce demoulding defects

**DOI:** 10.1007/s00170-024-13163-0

**Published:** 2024-02-19

**Authors:** Sana Zaki, Nan Zhang, Michael D. Gilchrist

**Affiliations:** School of Mechanical & Materials Engineering, University College Dublin, Belfield, Dublin 4, Ireland

**Keywords:** Microscale shaping and polishing, Green electrolyte, Nickel sulfamate, Electropolishing, Micro injection moulding, Hot embossing

## Abstract

Precise tooling is vital for defect-free production of micro injection moulded (μ-IM) or hot-embossed products. The demoulding stage of such moulding and forming processes poses a serious challenge to the integrity of thin miniature features because of friction, adhesion, and thermal stresses. Typically, micro moulds involve geometrically textured patterns or features such as linear ridges, pillars, channels, and holes, the characteristic dimensions of which range from 10 to 300 μm. Realistically complex mould designs, containing precision micro features (enhanced fillet radius and positive draft angle) and high surface quality, are presented in this work. Electropolishing based on forward pulse currents (PC) has been used to shape and polish Ni micro moulds that contain sets of micron-scaled linear ridges and star patterns in order to ease the separation of moulded polymeric parts from the metallic mould during ejection and demoulding. The use of forward pulsed currents improved the mould design by increasing the fillet radii and draft angle while keeping the surface roughness low and maintaining a good surface shine. An optimization study of forward PC using a green solution of nickel sulfamate varied EP times (0–70 min) and duty cycles (40, 50, 60, and 70%) at a process conditions of 2.8 V, 50 °C, and 250 rpm. The best topographical and morphological changes were observed for a typical microfluidic channel (*w* × *h*, 100 × 110 μm) with an EP time of 70 min and 50% duty cycle: fillet radius increased by 3.8 μm, draft angle by 3.3°, and the channel width reduced by 11.4% while surface roughness changed by 8.6% and surface shine improved by 48.9%. Experimental validation was performed using hot embossing wherein the electropolished Ni mould replicated the micro channels and star patterns in PMMA chips with notably fewer burrs, material pile up, and no feature distortion. Moreover, there was a reduction in the side wall roughness of micro channels in PDMS casting with electropolished Ni mould by 16%. Hence, this work presents a significant scientific contribution to improving the efficiency of micro mould tools and reduces the defects caused by friction and adhesion in replicated polymeric parts.

## Introduction

Polymer micro-nano fabrication processes are widely used for industrial and research applications in the fields of microfluidics [1], micro-optics [2, 3], and MEMs [4], amongst others. These forming and moulding processes include micro injection moulding [5], hot embossing [6], and nano imprinting [7]. Essentially, they involve replicating micro-nano scale features from a metal mould (Ni/composite) to a polymeric part. Micro injection moulding offers advantages including 3D geometries, low unit fabrication cost, and high volume throughput. However, the design, quality, and surface integrity of the mould tools are crucial for defect-free production. Friction [8], adhesion [9], and thermal stresses [10] are common factors that can cause defects and imperfections during the demoulding stage. Demoulding defects can include burrs, material pile-up, and feature distortion in a replicated polymer part. Typically, micro-nano mould tools have walls with rectangular profiles that have features smaller than 100 μm, which can increase the effects of friction and adhesion when ejecting a polymeric part from a mould. To improve mould tool design, positive draft angles and increased fillet radii are desirable since they serve to facilitate the separation of solidified polymer from a mould. Additionally, the use of 2D materials [11] and anti-stick coatings can enhance the surface integrity of produced parts: for example, ${MoS}_{2}$ [12, 13] ${WS}_{2}$, and *PTFE* [14] have all been shown to enhance the surface integrity of the produced parts. Micro injection moulded parts have miniature dimensions and correspondingly high surface to volume ratios, and greater injection pressures, all of which make the demoulding process more difficult than in conventional injection moulding.

Recent efforts have been made to improve mould design in order to ease demoulding. In recent studies on liquid composite moulding (LCM), [15] divided moulds into two parts to accommodate complex geometries. LCM is based on the use of a closed-mould without an autoclave and is used for manufacturing polymer matrix composite components. He machined the mould tools with a draft angle of 2° to facilitate demoulding of the cured component. Gülçür et al. [16] demonstrated the use of soft tooling for μ-IM machine using propylene resin for medical devices and microsystem components with a theoretical maximum shot size of 1.1 cm^3^ and a 5-mm injection piston. The mould tool was disc-shaped with a diameter of 13 mm and a thickness of 1.25 mm and had a high draft angle of 10° to facilitate part ejection. They managed to produce 100 parts consecutively with a cycle time of 29 s. In recent work by [17], the mouldability of additively manufactured attachments on a multipoint tool to produce carbon fibre reinforced plastics (CFRP) was examined. The additively manufactured attachment included a demoulding slope of 1–2.5° and a minimum rounding radius of 5 mm at the transition of the moulded parts in order to enhance their mouldability. Increasing the fillet radius was found to have a positive effect on mouldability. Hodgir et al. [18] examined the performance of various rapid casting methods, including Rapid Ice Investment Casting (RIIC), for creating complex shapes. The pattern making process for RIIC and others involved patterns with distinct fillet radii (inner and outer) and a 45° chamfer. The RIIC mould demonstrated superior performance and a smooth surface finish by incorporating draft angles and fillet radii in the mould pattern. However, in all of these moulds, the dimensions were notably larger than for micro-components and were typically in the range of millimetres in size; achieving the draft angles on these moulds was quite feasible. For micro moulds, however, the fabrication of draft angles is challenging because of the physical dimensions of machine tools.

Electropolishing is a non-contact electrochemical process that effectively removes burrs [19], asperities [20], and other impurities from metal surfaces [21]. Unlike other microfabrication techniques such as metal-assisted chemical etching [22], laser polishing [23], and deep reactive ion etching [24], electropolishing stands out because it removes material based on current density distribution [25], while other processes are limited by their anisotropic nature. As a result, electropolished metal surfaces become level and bright and have low surface roughness, while metals with micro features develop rounded edges, thinner features, and changes in draft angles. A typical electropolishing cell comprises an anode (working metal), a cathode (counter electrode), and a magnetic stirrer immersed in the electrolyte. The anode and cathode are connected to a power supply, which can be either DC or AC, to polarize the cell. Redox reactions take place at both electrodes, with oxidation occurring at the anode and reduction at the cathode. The metal (anode) dissolves in the solution by forming ions. This process was initially explained by Jacquet and Elmore. Jacquet [26] elucidated the formation of a resistive ionic layer at an anode, which facilitates the formation and movement of metal ions due to the uneven surface. Elmore [27], on the other hand, described the diffusion of anions through a concentration gradient near the anode. Both theories explain the reduction of surface roughness and the brightening of metal surfaces in acidic solutions.

During electropolishing, the metal being treated dissolves in the solution through diffusion/migration and eventually develops an oxide layer. The kinetics of electropolishing are illustrated by the following Eqs. (1–3):

1 $${\text{Anode}}: {M}^{o}\to {M}^{z+}+z{e}^{-}$$

2 $${\text{Cathode}}: 2{H}^{+}+2{e}^{-}\to {H}_{2}$$

3 $$\mathrm{Formation\;of\;oxide\;layer}: Me+2{OH}^{-}\to MeO+{H}_{2}O+2{e}^{-}$$

**Fig. 1:**
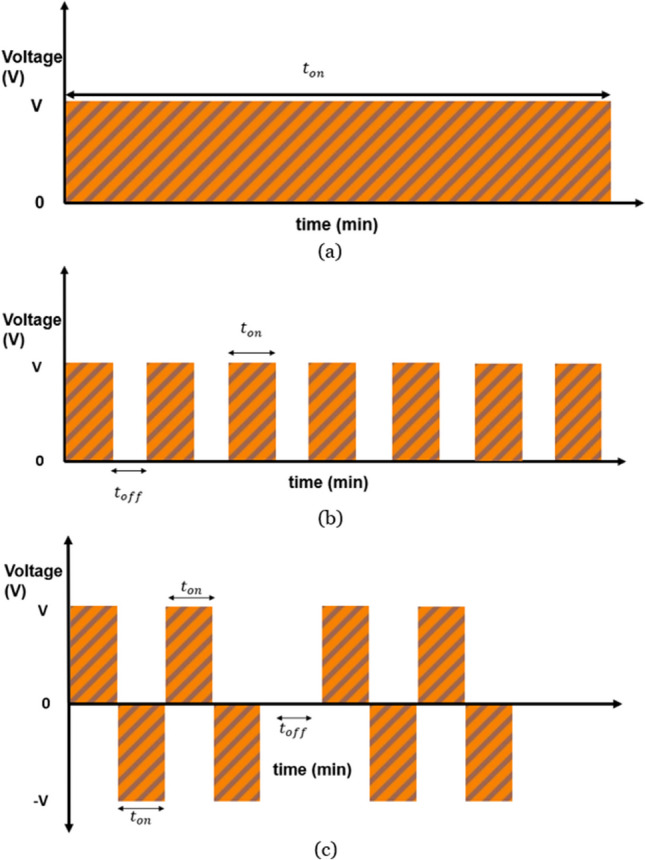
Three waveforms for electropolishing: **a** direct current, **b** forward pulsed current, **c** pulse/pulse reverse current

The phenomenon of electropolishing has been explained through several other theories that describe concepts such as acceptor ions [28], passivation (strongly passivating metals) [29], and adsorption (noble/precious metals) [30] for certain metals. Additionally, there are various waveforms used in electropolishing, including direct current (DC) [31], forward pulse current (PC) [31], and pulse/pulse reverse current (PPR) [32]. DC is a typical waveform that was commonly used by early researchers in the field. It explains material removal and surface levelling through anodic dissolution up to the hydrodynamic limit. Both PC and PPR are pulsating wave forms with positive, negative, and off cycles. They feature high rapid anodic pulse currents and a higher material removal rate, offering advantages over normal DC waveforms. The forward pulse removes material [33], the off-pulse assists in the removal of by-products and ensures a fresh circulation of electrolyte, and the reverse pulse reduces or removes any formed oxide layer [34]. Figure 1 illustrates the three waveforms used in electropolishing. PPR is used for strongly passivating metals such as stainless steel, Inconel, titanium, and niobium alloys [35–37], while PC is used for non-passivating metals such as nickel, tungsten, and aluminium alloys [38–40].

A pulsed wave is defined by several parameters including peak voltage (V), duty cycle (DC), and frequency (f) as shown in Eqs. (4 and 5). Any pulsed wave form is dependent on the pulse being on (either positive or negative), being off, and the total time:

4 $$D.C=\frac{{t}_{on}+ {t}_{off}}{{t}_{t}}$$

5 $$f= \frac{1}{{t}_{t}}$$

where “${t}_{on}$” is the pulse on time, “${t}_{off}$” is the pulse off time, and “${t}_{t}$” is the total pulse time.

**Fig. 2:**
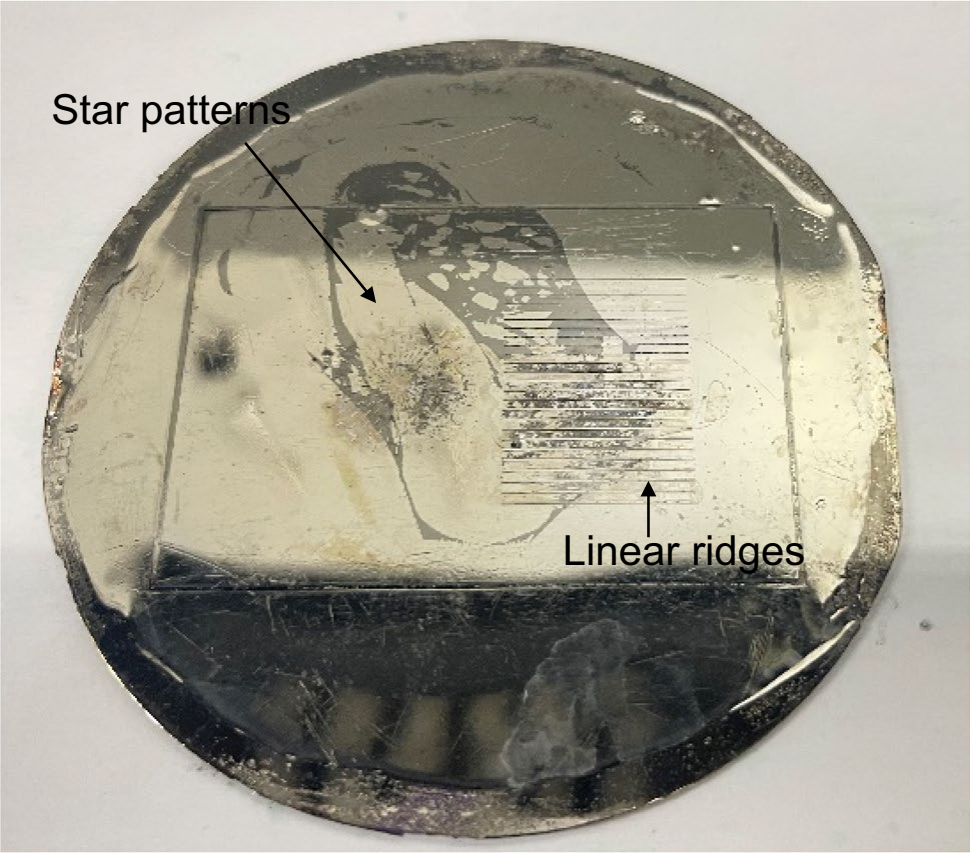
Ni mould with micro linear and star patterns manufactured through a typical dry etching, electroforming, and moulding (DEEFM) process combining silicon lithography and an electroforming process. NOTE: Diameter of wafer is 4 in

Inspired by the aforementioned literature, the present work proposes a solution to minimizing or avoiding demoulding defects. The aim is to combine the effects of shaping and polishing of micro-Ni moulds, which are fabricated through silicon dry etching, metallization, and electroforming (DEEFM). A set of multi-linear ridges and star patterns, as shown in Fig. 2, have been designed as representative of typical features commonly used in micro moulding. The dimensions of the micro features range from 30 to 150 μm in width and 80–110 μm in height. Such features are commonly found in microfluidic devices. The process of electropolishing is used to induce topographic and morphological changes on the Ni moulds. This includes achieving a positive draft angle, increased fillet radius, and reduction in feature width, as well as reducing surface roughness and improving surface shine. This combination of modifications significantly mitigate the demoulding issues due to adhesion, friction, and thermal stresses at the interface between the Ni mould and polymer part. To validate the effectiveness of the shaping and polishing process, a study was conducted by observing the side wall roughness using PDMS casting and by producing PMMA chips with linear and star-patterned Ni moulds by means of hot embossing. The results of this investigation demonstrate an improvement in the efficiency of the Ni mould tools following the shaping and polishing process. For electropolishing, the Ni mould tools were treated in a green solution of nickel sulfamate using forward pulsed currents, since Ni is not a strongly passivating metal. In preliminary studies conducted by the authors (unpublished), optimal shaping conditions were found using a voltage of 3 V for 15 min at a temperature of 50 °C. The best polishing results, however, were achieved using a voltage of 2.8 V for 2.5 min at a temperature of 50 °C in the same nickel sulfamate solution. To combine the effects of shaping and polishing, an optimization study on forward pulsed currents was conducted, and is the basis of this present paper. The impact of electropolishing at various EP times (0, 25, 40, 55, and 70 min) and duty cycles (40%, 50%, 60%, and 70%) was examined to achieve the desired topographic changes, such as increased fillet radii, positive draft angles, and feature width reduction, as well as morphological changes, including reduced surface roughness (Sa) and increased surface shine. The optimized parameters of pulsed electropolishing provided significant design changes in the mould tools, effectively reducing defects such as burrs, material pile-up, and feature distortions caused by friction and adhesion between the metal mould and polymer chip.

## Materials and methods

### Ni wafer with linear ridges and star patterns

A pure Ni (99.5%) mould as shown above in Fig. 2 was manufactured using dry etching, electroforming, and moulding based on silicon lithography [41] and electroforming [42]. Two design features were patterned on 4-inch silicon wafers using ultraviolet lithography, deep reactive ion etching (DRIE), followed by a conductive layer of titanium (50 nm) and nickel vanadium (200 nm), which was electroformed and then etched in a solution of potassium hydroxide (KOH). Feature dimensions of linear ridges and star patterns varied in width from 20 to 200 μm and height from 80 to 110 μm. In order to perform the pulsed current experiments, samples were cut from wafer with nominal size of 115 mm^2^ having linear ridges. While performing hot embossing experiments, the complete Ni mould with linear and star patterns was utilized.

### Sample preparation

To enhance surface reactivity, all Ni moulds underwent an ultrasonic bath in deionized water for 5 min, followed by acetone for 5 min. To insulate the unpolished surface, the back sides of the samples were coated with an aerosol conformal coating, which maintained an operating temperature range of +200 to −70 °C.

### Characterization of samples and electrolyte

The feature topology and surface morphology of the samples were assessed using white light interferometry (NP Flex Bruker, USA) at a magnification of ×20. Various filters were applied to differentiate feature form and roughness, including statistic, data restoration, tilt removal, and Gaussian regression. A long wavelength of 0.01–0.03 mm was used for 2D profile analysis, while a short wavelength of 0.025 mm was used to assess surface roughness. The 3D profiles of the microfeatures were analyzed to observe changes in feature shape and surface roughness, while the 2D profiles were examined to identify geometric changes such as fillet radii and draft angle. Changes in friction, adhesion, and surface defects and features on the PMMA chips that were produced from electropolished Ni moulds were evaluated by using a benchtop SEM TM-4000. The SEM allowed for imaging using standard electron beams (SE) and backscattered electron beams (BSE) at high accelerating voltages (5, 15, and 20 kV) and magnifications ranging from ×25 to ×250,000. The change in gloss of the samples after electropolishing was measured using a YG60 60° accurate Gloss Metre (Shenzhen 3nh Technology Co, China). Additionally, a digital microscope (AmScope MU1000 with 10MP) allowed for manufacturing defects on PMMA chips to be observed directly and facilitated a comparison between those produced from untreated and electropolished Ni moulds.

**Fig. 3:**
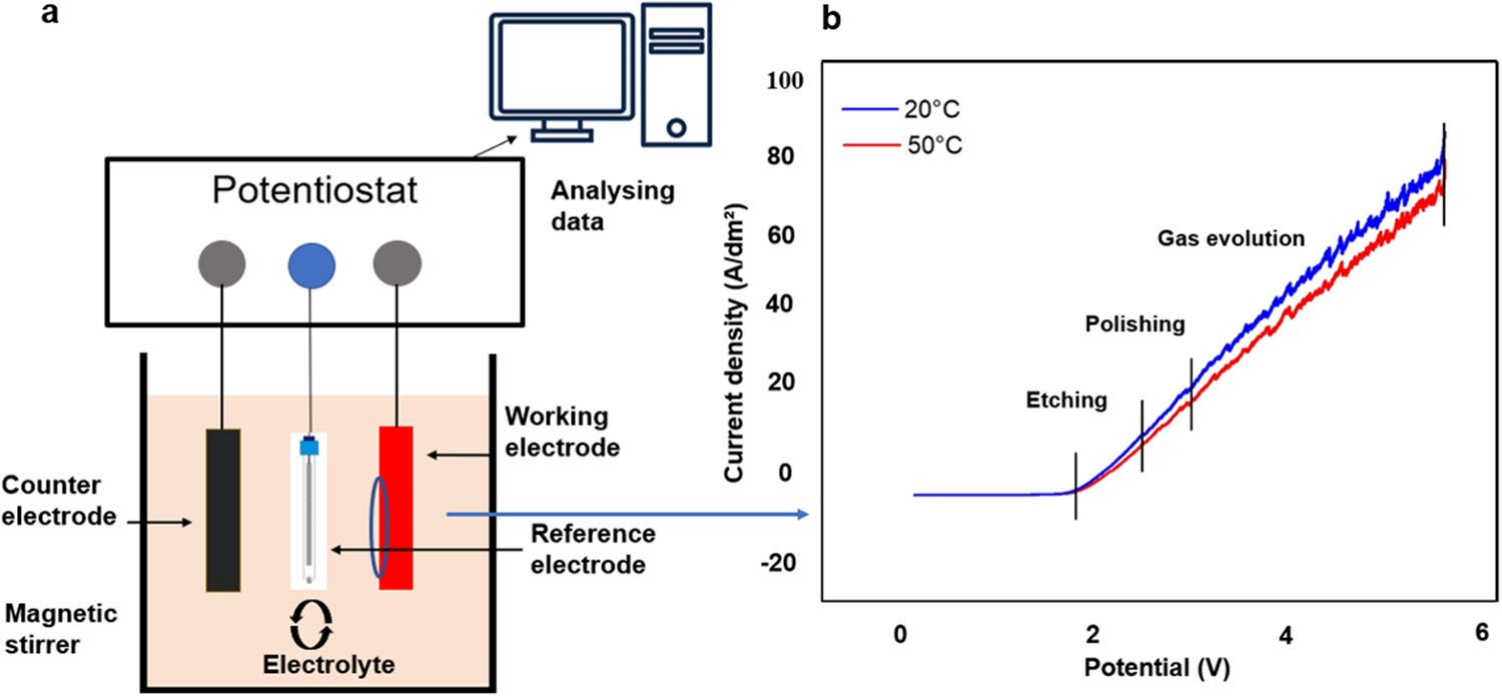
Potentiodynamic polarization behaviour of Ni mould in the solution of nickel sulfamate. **a** Three electrode standard polarization cell. **b** Polarization curve of Ni mould showing regions of etching, polishing, and gas evolution/passivation

An environmentally friendly electrolyte [43], nickel sulfamate solution, was chosen instead of conventional acids such as sulphuric and phosphoric acid [44]. The nickel sulfamate solution was characterized using a CS310 electrochemical workstation (Wuhan CorrTest, China). Linear scanning voltammetry (LSV) was conducted in a three-electrode standard cell. The Ni mould sample served as the anode, 314 SS as the cathode, and Ag/AgCl as the reference electrode. The LSV involved sweeping the potential from 6 to 0 V at a scanning speed of 10 mV/s. The potentiodynamic curve of Ni in the nickel sulfamate solution is depicted in Fig. 3, illustrating three distinct regions of etching, polishing, and gas evolution.

### Electropolishing cell

**Fig. 4:**
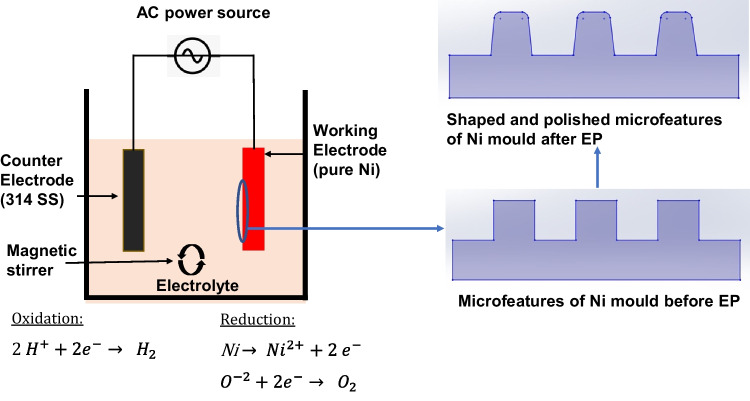
Electropolishing cell for precision shaping of microfeatures and polishing of Ni micro mould tools

A pulse reverse power supply (type pe86CB-20–5-25-S/GD, plating electronic GmbH, Germany) was used for the electropolishing process. This power supply offers both forward and reverse operation and can be programmed to generate complex waveforms with 16-step programming. It has a maximum rating of 20 V for voltage and 25 A for current. The electropolishing cell consisted of a Ni mould with microfeatures serving as the anode, and a flat SS cathode submerged in the green solution of nickel sulfamate. Both the anode and cathode were connected to an AC power supply. To prepare the electrolyte solution, a 2 M nickel sulfamate solution (H_4_N_2_NiO_6_S_2_, Ampere Galvanik Germany) was created by adding boric acid (20–30 g) and a wetting agent (1 ml). Electropolishing was carried out using forward pulsed currents, where the Ni mould (anode) dissolved in the nickel sulfamate solution at various frequencies, resulting in the shaping and polishing of microfeatures, as shown in Fig. 4. During the process, oxidation reactions occurred at the anode, leading to the liberation of oxygen, while reduction took place at the cathode, producing hydrogen gas.

The electrochemical cell provides an explanation of the topological and morphological changes that occur after electropolishing.

### Optimization of forward pulse currents (PC)

A systematic study was conducted using forward pulsed currents for electropolishing, with variations in time and duty cycles as outlined in Tables 1 and 2.

**Table 1:** Variation in EP time

Parameter	Variation
Electropolishing conditions	*I* = 0.01–0.03 A, ${V}_{{\text{m}}}$ = 2.8 V, ${V}_{{\text{f}}}$ = 2.0–2.2 V, *T* = 50 °C, *m* = 250 rpm
Total EP time	25, 40, 55, and 70 min
Anodic time (${t}_{on}$)	100 ms
Off time (${t}_{off}$)	100 ms
Frequency (Hz)	5
Duty cycle	50%

**Table 2:** Variation in duty cycle

Parameter	Variation
Electropolishing conditions	*I* = 0.01–0.03 A, ${V}_{{\text{m}}}$ = 2.8 V, ${V}_{{\text{f}}}$ =2.0–2.2 V, *T* = 50 °C, *m* = 250 rpm, *t* = 70 min
Anodic time (${t}_{on}$)	80, 100, 180, and 140 ms
Off time (${t}_{off}$)	120, 100, 120, and 60 ms
Frequency (Hz)	5, 5, 3.33, 5
Duty cycle	40%, 50%, 60%, and 70%

**Fig. 5:**
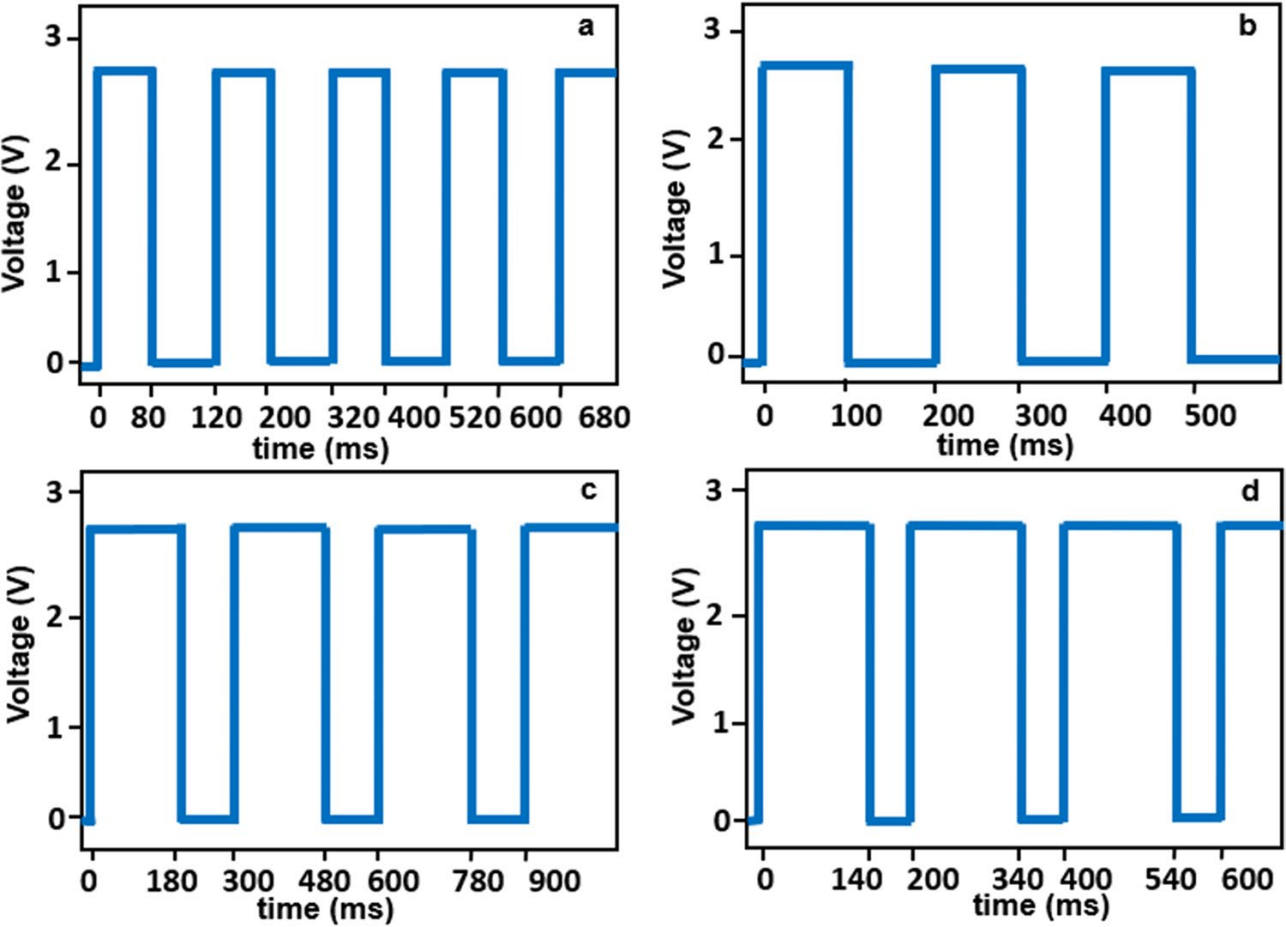
Electropolishing of Ni micro mould tools at various duty cycles: **a** 40%, **b** 50%, **c** 60%, and **d** 70%

The experiments were conducted in two stages: a time-based study and a duty cycle-based study. In the time-based study (Table 1), electropolishing was performed with a 50% duty cycle. The current was set between 0.01 and 0.03 A to ensure that the maximum voltage remained below 2.8 V. The fluctuating voltage (${V}_{{\text{f}}}$) observed for the set current was recorded, and the time was increased in intervals of 15 min from 0 to 70 min. In the duty cycle-based study (Table 2), electropolishing was performed at various duty cycles (40, 50, 60, and 70%). Similarly, the current was set between 0.01 and 0.03 A to maintain the maximum voltage below 2.8 V, and the fluctuating voltage (${V}_{{\text{f}}}$) was recorded. The variations in the on and off pulse times can be observed in Fig. 5.

### Surface morphology characterization

**Fig. 6:**
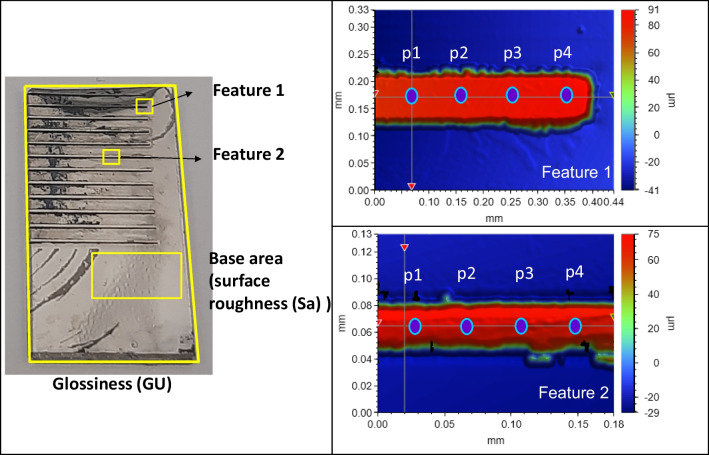
Measurement of surface morphology and topography

The Ni mould samples with linear ridges were characterized at four different areas, as illustrated in Fig. 6. Dimensional details of linear ridges for the duty cycle study are given in Table 3. Surface morphology was evaluated using two measurements: surface roughness (Sa) and overall shine (GU). The focus for the change in surface roughness was a small flat area beneath the linear micro ridges. Surface shine was observed across the entire sample, including the micro patterns and flat surface. Feature topography was assessed through three measurements: change in draft angle, increase in fillet radius, and width reduction. These aspects are discussed in detail below in Section 3.1.2.

**Table 3:** Detailed dimensions of micro features

Samples	Width (μm)	Height (μm)
Sample 1 (40% DC)		
Feature 1	100	110
Feature 2	150	110
Sample 2 (50% DC)		
Feature 1	100	110
Feature 2	35	100
Sample 3 (60% DC)		
Feature 1	100	100
Feature 2	40	100
Sample 4 (70% DC)		
Feature 1	100	100
Feature 2	150	100

It should be noted that all samples initially had a conductive layer coating, which was subsequently removed during the pulsed electropolishing process.

### Feature topography characterization

**Fig. 7:**
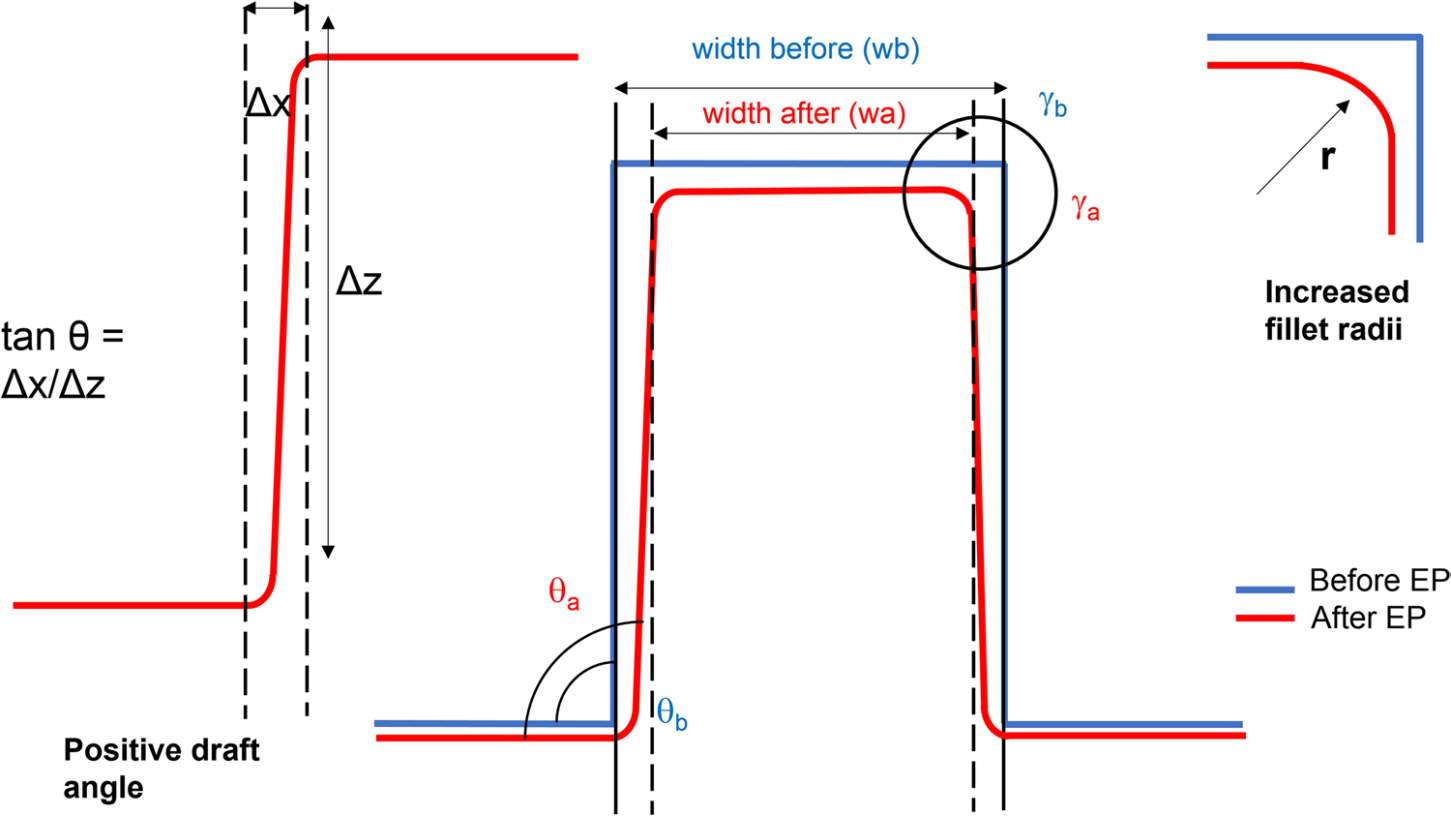
Measurement of draft angle and fillet radii

The examination of feature topography involved analyzing the changes in geometry, including fillet radius, draft angle, and width reduction, of the Ni mould after electropolishing. Two micro features were observed on each sample, with one feature located at the right corner and the other near the middle of the ridge, as depicted in Fig. 7. In the vision64 software, 2D graphs were generated using the white light interferometer to visualize the changes in the two micro features. Additionally, four points were selected along the length of each ridge to observe variations in fillet radius, draft angle, and width reduction before and after electropolishing. A schematic in Fig. 7 illustrates all the geometric changes that were observed for the samples. The measurements for draft angle were conducted in vision64, while the analysis of fillet radii was performed in Matlab using a curve fitting technique.

### Side wall roughness characterization

**Fig. 8:**
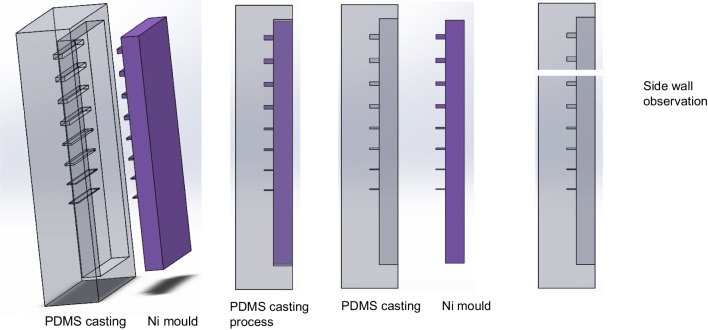
Measurement of side wall roughness using PDMS casting method (SLYGARD 184 Silicone elastomer kit) with the Ni mould having linear micro ridges

An electropolishing (EP) validation study was conducted on the Ni moulds to observe the changes in the roughness of the side wall. A PDMS casting was taken of the Ni mould with linear micro ridges in order to quantify the surface roughness. SYLGARD 184 Silicone elastomer, which consisted of an elastomer base and an elastomer curing agent, was used for this purpose. The Ni mould was placed inside a sealed container, and a mixture of the elastomer base and curing agent was applied in a ratio of 10:1 after removing air bubbles via vacuuming. The mixture was then cured at 60 °C for 24 h, following which the metal mould was separated from the PDMS part. The micro patterns of the Ni mould were replicated on the PDMS casting, forming micro channels. For the side wall measurement, the PDMS casting was sliced along the micro feature wall, as illustrated in Fig. 8. The sectioned PDMS wall was subsequently sputter coated with a layer of gold, and its side wall roughness was examined using a 3D profilometer.

### Hot embossing to produce polymeric chips

**Fig. 9:**
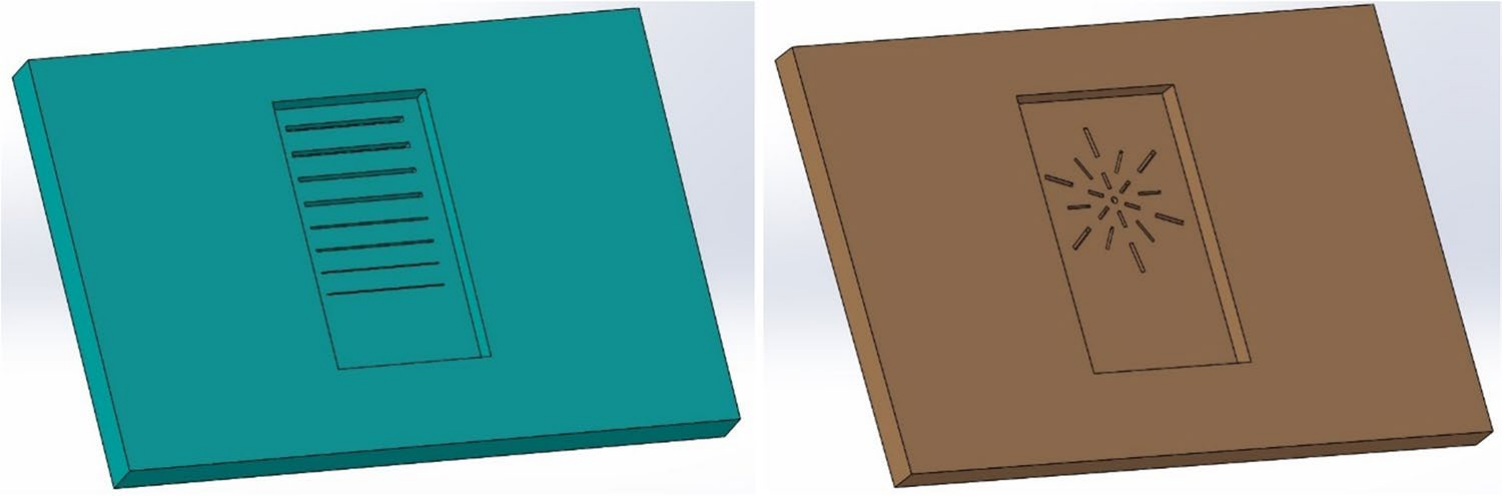
Making PMMA polymeric chips using hot embossing with linear and star shaped patterns for validation of electropolished Ni moulds

Hot embossing was used to fabricate polymer chips using both treated and untreated Ni moulds. Two types of patterned nickel moulds were used, one featuring linear micro ridges and the other containing star patterns, as indicated in Fig. 9. The PMMA chips were produced by using the Ni moulds for hot embossing under specific parameter settings, namely a temperature of 115 °C for the linear micro channels and 90 °C for the star patterns, with gradual time intervals ranging from 2 to 7 min. This validation process aimed to assess the reduction in friction and adhesion defects such as burrs, material pile up, and feature distortions on the replicated polymeric chips after the electropolishing process.

## Results and discussion

### Shaping and electropolishing of linear micro ridges

#### Change in feature topography by varying EP time

**Fig. 10:**
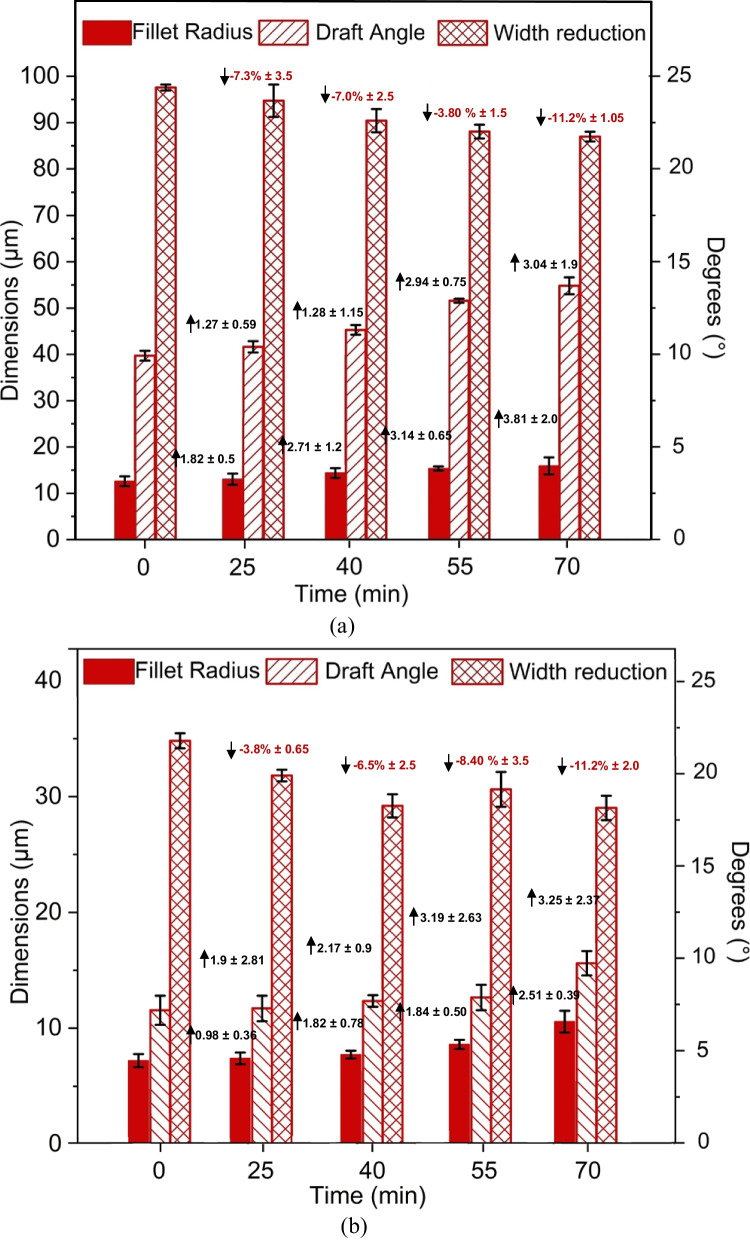
Feature topography of Ni micro mould tool electropolished at 50% duty cycle by varying electropolishing time. **a** Feature 1. **b** Feature 2

An investigation was undertaken into the effect of electropolishing (EP) time on the geometric and morphological changes of Ni moulds. The parameters of pulsed electropolishing are outlined in Table 1, where electropolishing was performed at a fluctuating voltage of 2–2.2 V, a temperature of 50 °C, current of 0.01–0.03 A, and a magnetic agitation rate of 250 rpm for different durations of 25, 40, 55, and 70 min. Increasing the EP time resulted in more pronounced shaping and forming of micro ridges, as evidenced by the increased fillet radii, positive changes in draft angle, and reduced feature widths. The geometric changes for two micro features are illustrated in Fig. 10. Feature 1 (size, *w* × *h* = 100 × 110 μm) exhibited shaping and forming with an increased fillet radius (14–17.8 μm), positive change in draft angle (11.6–14.9°), and width reduction (97.6–86.9 μm). Similarly, Feature 2 (size, *w* × *h* = 35 × 100 μm) also demonstrated shaping and forming with an increased fillet radius (4.9–7.7 μm), positive change in draft angle (7.2–10.6°), and width reduction (34.8–29.0 μm).

#### Change in surface morphology by varying EP time

**Fig. 11:**
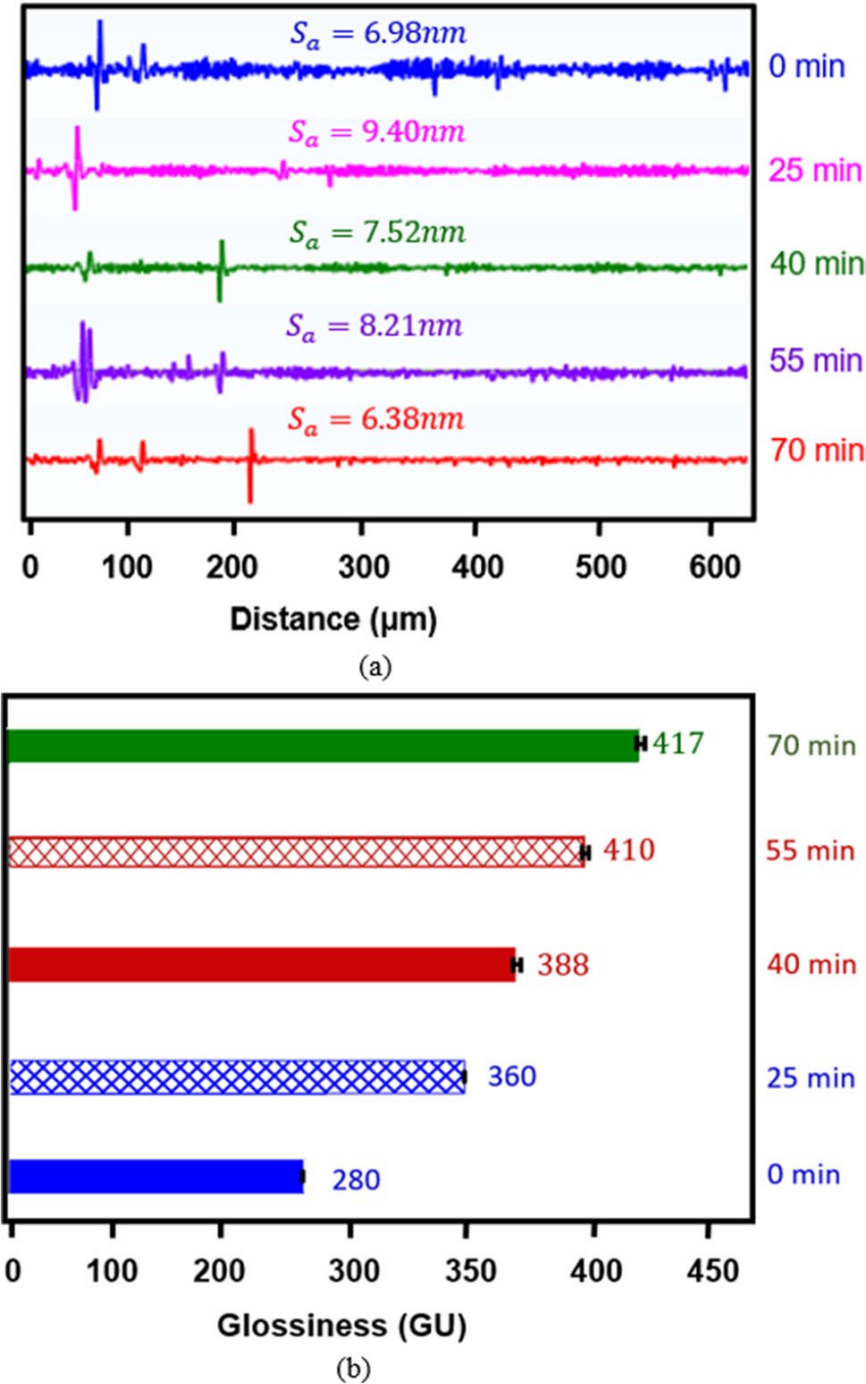
Surface roughness (**a**) and surface gloss (**b**) of Ni micro mould tool electropolished at 50% duty cycle by varying electropolishing time

The surface morphology of Ni mould tools has been examined from two perspectives: surface roughness (Sa) and surface shine (GU). Both of these aspects are crucial for achieving defect-free production of polymeric chips for various microfluidic devices. Figure 11 illustrates the changes in surface roughness and surface shine for the Ni mould as the electropolishing (EP) time increases at a 50% duty cycle. Initially, as the metal begins to dissolve in the solution, the surface roughness increases slightly. However, as the EP time progresses, the surface becomes more level and undergoes micro polishing, resulting in a slightly lower Sa value compared to the initial state. On the other hand, throughout the EP process, the surface shine continuously increases from 280 to 417 GU.

#### Change in feature topography at various duty cycles

**Fig. 12:**
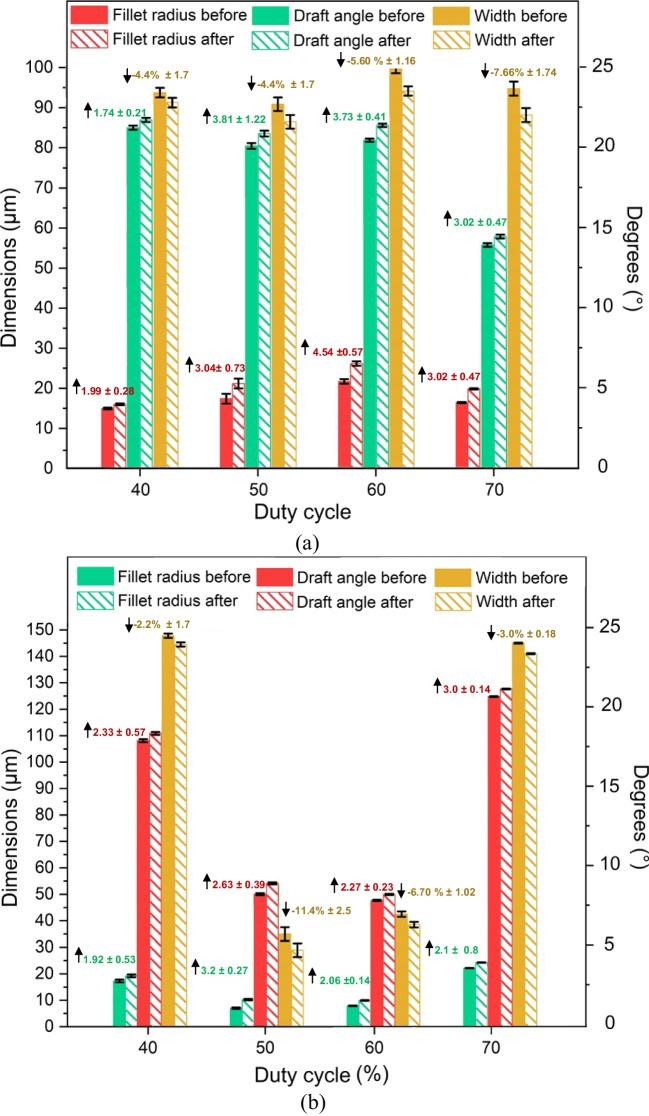
Feature topography of Ni micro mould tool electropolished at various duty cycles. **a** Feature 1. **b** Feature 2

An optimization study was conducted based on duty cycle, with the parameters detailed in Table 2. Four duty cycles (40%, 50%, 60%, and 70%) were selected to evaluate the effectiveness of shaping and polishing using forward pulse currents in electropolishing (EP). The geometric changes observed are depicted in Fig. 12, where two features show a clear increase in fillet radius, draft angle, and width reduction after electropolishing. Feature 1 (size, *w* × *h* = 100 × 110 μm) undergoes shaping and forming, resulting in changes in fillet radii (+ 1.99–4.54 μm), positive draft angle (+1.74–3.81°), and width reduction (−4.4–7.66%). Similarly, Feature 2 (size, *w* × *h* = 150 × 100 μm, 35 × 100 μm, 45 × 100 μm, and 145 × 100 μm) is also shaped and formed to have an increase in fillet radii (+1.92–3.2 μm), positive draft angles (2.33–3.00°), and width reductions (−2.2–11.4%).

The best overall shaping results, as characterized by an increased fillet radius, positive draft angle, and width reduction, were observed for the 50% duty cycle.

#### Change in surface morphology at various duty cycles

**Fig. 13:**
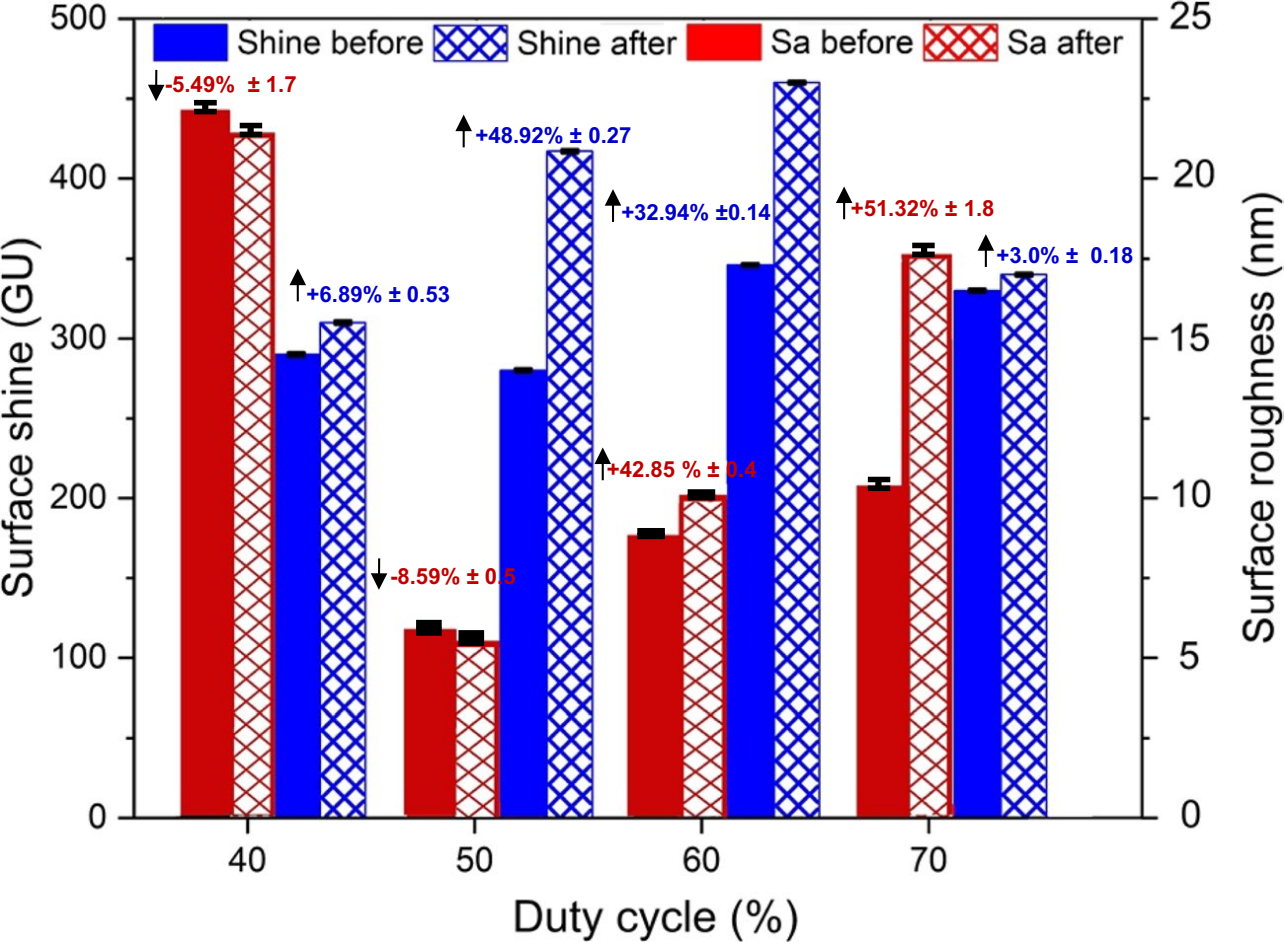
Surface morphology indicating change in surface roughness and shine of Ni micro mould tool electropolished at various duty cycles

The surface morphology of the Ni mould was examined in terms of changes in surface roughness (Sa) and shine (GU). The results of the duty cycle study, shown in Fig. 13, demonstrate variations in surface roughness and shine as the on and off cycle times vary. Notably, there is a consistent increase in surface shine ranging from 3.0 to 48.9% across all duty cycles. In contrast, the surface roughness decreases from 5.5 to 8.5% for the initial two duty cycles (40% and 50%), while it increases from 42.8 to 51.3% for the remaining two duty cycles (60% and 70%). When developing Ni moulds, it is crucial to maintain a low surface roughness (<50 nm) while also maintaining or enhancing surface shine. A mould surface with low roughness and a glossy appearance can effectively reduce demoulding defects in the micro injection moulding process.

### Optimized parameters for shaping and polishing of Ni moulds

**Fig. 14:**
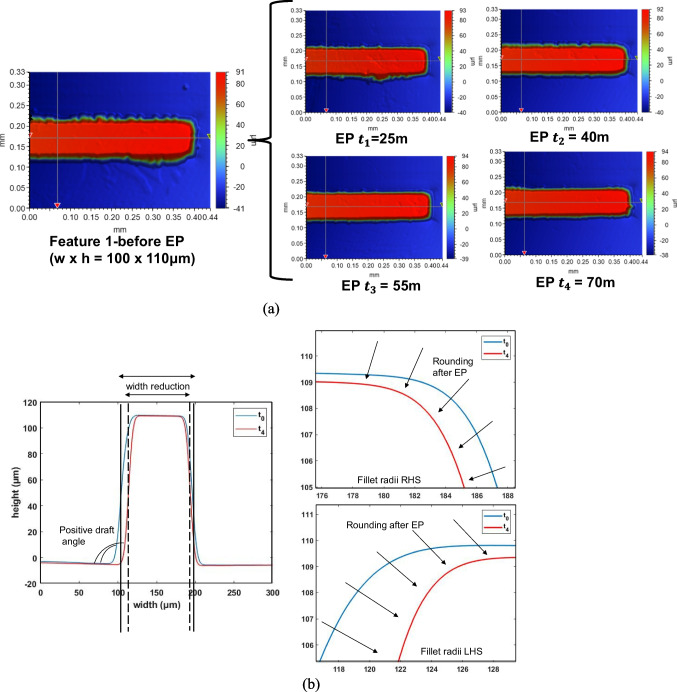
3D and 2D profile data of micro Feature 1 showing geometric changes with the increase in EP time at a duty cycle of 50%. **a** 3D profile of microfeature at different time intervals 0–70 min. **b** 2D overlay of microfeature before and after electropolishing for 70 min with all geometric changes (i.e. increase in fillet radii, positive draft angle and width reduction)

An optimization study was conducted to determine the best parameters for shaping and polishing Ni micro moulds by varying two factors: electropolishing time and duty cycle. The results indicated that the optimal parameters for improving the mould design were an electropolishing time of 70 min and a duty cycle of 50%. Increasing the electropolishing time led to desirable geometric changes such as increased fillet radii, draft angles, and width reductions due to increased material removal. Similarly, increasing the duty cycle enhanced the geometric changes, but it also had a negative impact on surface quality, especially at higher duty cycles (60% and 70%). In the micro injection moulding process, it is typically required to have reduced surface roughness (Sa) and improved surface shine for better efficiency and performance. Figure 14 illustrates the optimized results for the micro feature, which was gradually electropolished over a period of 0 to 70 min at a 50% duty cycle. The geometric changes clearly improved the mould design. These findings were further validated through measurements of the side wall roughness and the subsequent production of PMMA polymer chips using both untreated and electropolished Ni moulds, as discussed below.

### Validation of electropolishing and shaping of micro mould tools

#### Measurement of side wall roughness

**Fig. 15:**
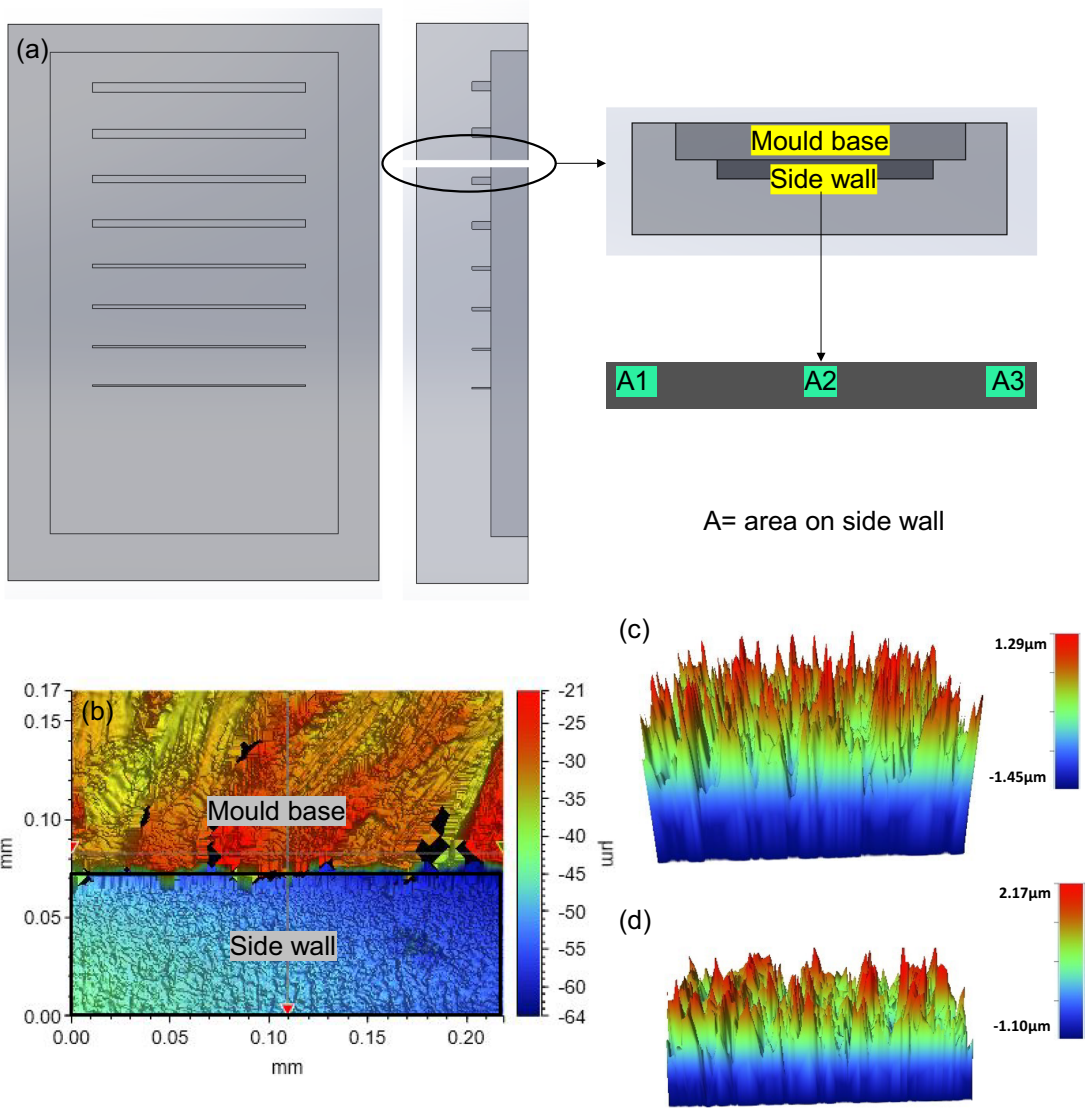
PDMS casting of Ni micro mould features for the measurement of side wall. **a** 3D schematic of PDMS casting explaining the cutting of PDMS sample along the wall at three different areas. **b** Sectioned piece of side wall showing the mould and micro wall under white light interferometer. **c** Surface roughness of side wall before EP. **d** Surface roughness of side wall after EP

After successfully implementing pulsed current electropolishing to improve the mould design and surface quality, two validation experiments were conducted. The first experiment involved measuring the side wall roughness, while the second experiment focused on reducing friction and adhesion forces. In order to measure side wall roughness, a PDMS casting method was used. This started with using the Ni mould tool with linear micro ridges to cast patterns into PDMS (casting time 24 h), both before and after electropolishing. Specifically, a linear micro ridge with dimensions of 100 × 110 μm was selected for analysis. The PDMS cast part was sliced, as depicted in Fig. 15, to expose the side wall of the microfeature. Subsequently, the PDMS part was sputter coated with a layer of gold and examined using a 3D profilometer to assess the side wall roughness. The results summarized in Table 4 indicate a noticeable reduction in the roughness of the side wall after electropolishing. Three areas were measured on the side wall of the PDMS casting so as to replicate the Ni mould micro wall: there is a clear reduction in surface roughness after electropolishing brings the side wall roughness down by an average of 16%. This is significant validation for the implementation of better tool performance and this improvement in surface quality is expected to enhance the efficiency of the mould tool in the μ-IM process.

**Table 4:** Surface roughness noted on various areas of the micro linear wall

Area	Sa before EP (nm)	Sa after EP (nm)	Percentage reduction (%)
1	265.9	229	14
2	230	199	13
3	233	182	22

Average reduction in roughness = 16%

#### PMMA chips produced using hot embossing

**Fig. 16:**
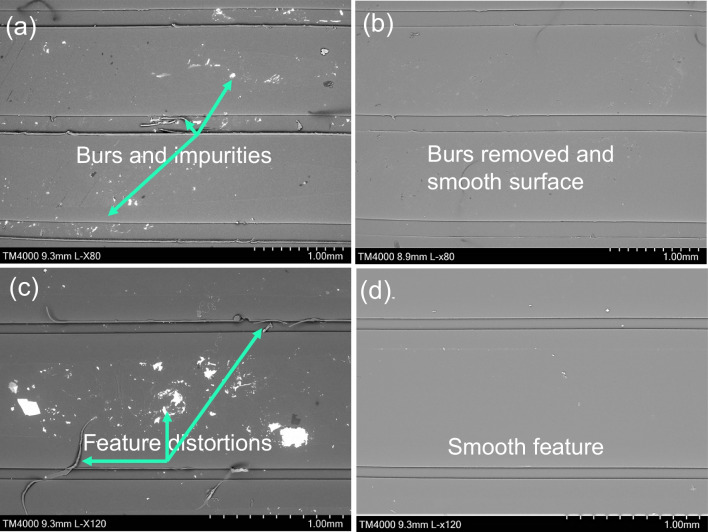
SEM images of linear PMMA chips showing common adhesion and friction effects after hot embossing produced with polished and unpolished Ni mould. **a**, **c** SEM image of PMMA chip produced using untreated Ni mould. **b**, **d** SEM image of PMMA chip produced using electropolished Ni mould

The electropolished Ni moulds were further validated by using them to produce polymer chips made of PMMA, incorporating both linear micro ridges and micro star patterns by means of hot embossing. The aim was to observe any manufacturing defects such as burrs, material pile up, or feature distortions that might be caused by friction and adhesion. Figure 16 shows the replicated linear micro ridges in the PMMA chips, achieved through hot embossing using the Ni moulds. Observing the PMMA chips, it can be seen that some manufacturing defects in the form of feature distortions and material pile up occurred as a result of friction and adhesion within the replicated micro channels. To illustrate the presence of a conductive layer that might subsequently be removed during electropolishing, images of both untreated and electropolished Ni moulds have been included.

**Fig. 17:**
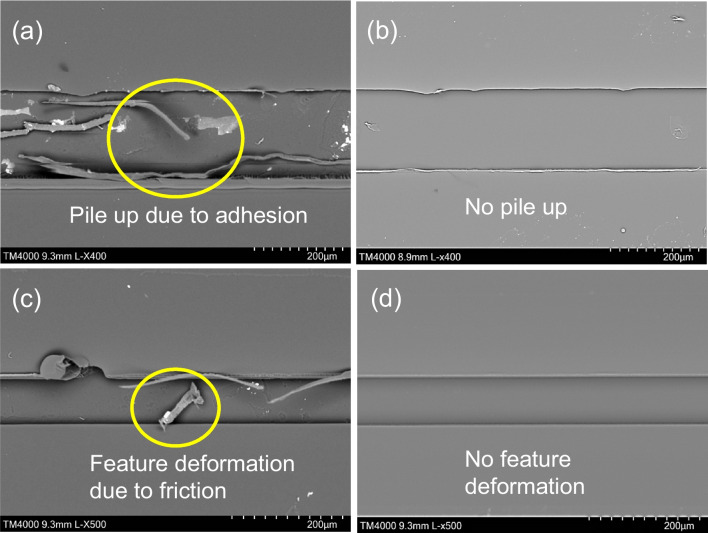
SEM images of linear PMMA chips produced with polished and unpolished Ni mould with common defects due to friction and adhesion. **a**, **c** SEM image of PMMA chips produced using untreated Ni mould having burrs and pile up issue. **b**, **d** SEM image of PMMA chips produced using electropolished Ni mould with burs and pile up removed

Close examination of Fig. 17 highlights the replicated microchannels, specifically the occurrence of pile up and feature distortion resulting from friction and adhesion between the Ni mould and PMMA chip. These issues, however, were effectively addressed through the process of electropolishing, as it is seen that the electropolishing treatment successfully eliminated both feature distortion and material pile up problems, and managed to improve replication quality, which would subsequently enhance performance of the microchannels.

**Fig. 18:**
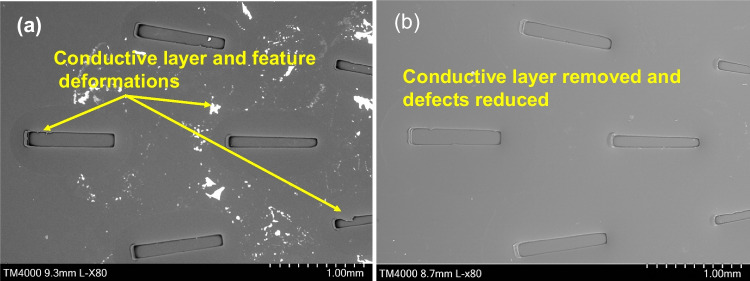
SEM images of star PMMA chips produced with polished and unpolished Ni mould with common defects due to friction and adhesion. **a** SEM image of PMMA chip produced using untreated Ni mould with conductive layer. **b** SEM image of PMMA chip produced using electropolished Ni mould with conductive layer removed

Similar to the Ni mould with a linear pattern, a star-patterned Ni mould was used to fabricate PMMA chips. The purpose of this validation was to assess the reduction in manufacturing defects achieved through the electropolishing process. Figure 18 presents the PMMA chips featuring the star patterns, produced using both the untreated and electropolished Ni moulds.

**Fig. 19:**
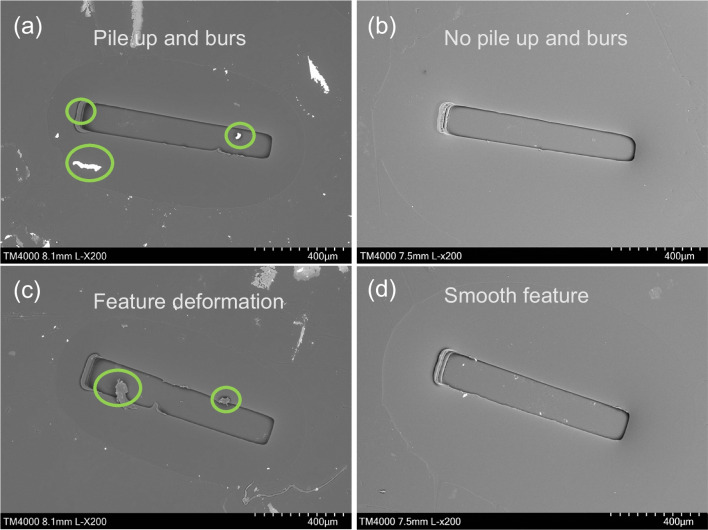
SEM images of star PMMA chips produced with polished and unpolished Ni mould with common defects due to friction and adhesion. **a**, **c** SEM image of PMMA chip produced using untreated Ni mould. **b**, **d** SEM image of PMMA chip produced using electropolished Ni mould

The electropolishing process effectively reduced the manufacturing defects in the star-patterned PMMA chips. Initially, the Ni mould possessed a conductive layer that was replicated onto the surface of the polymeric part. However, after undergoing electropolishing, the detrimental effects of adhesion and friction were significantly diminished. As a result, the production of defect-free PMMA chips featuring both linear channels and star patterns was achieved (Fig. 19).

## Conclusions

In this research, the combination of shaping and polishing techniques using pulsed current electropolishing (EP) was investigated. An optimization study was conducted to determine the optimal EP time and duty cycle that would result in the most favourable topographic and morphological changes in a Ni mould tool, with the aim of reducing demoulding defects. The optimized parameters were then validated through a series of experiments using Ni mould tools featuring linear micro ridges and star patterns. The validation study involved measuring the side wall roughness and evaluating the quality of the polymeric chips produced. The results demonstrated a significant reduction in friction and adhesion between the metal mould and the polymer part. This successful validation of using electropolishing as a surface finishing process confirmed its effectiveness in improving the quality of metal mould tools for subsequent use in mass manufacturing moulding and forming processes. Based on the experimental findings and the validation study, the following conclusions can be drawn:

- The optimization study on pulsed currents revealed that the best topography, characterized by an increase in fillet radius, positive draft angle, and width reduction, along with improved surface morphology, including reduced surface roughness and enhanced shine, could be achieved for Ni mould tools. The optimal parameters were identified as a duty cycle of 50% and an EP time of 70 min. The optimized shaping resulted in an increased fillet radius of 3.8 μm, a positive draft angle of 3.3°, and a width reduction of 11.4%. Moreover, the optimized polishing process led to a reduction of 8.6% in surface roughness (Sa) and a significant improvement in surface shine, namely 48.9%.
- The Ni mould tools underwent shaping under different duty cycles, namely 40%, 50%, 60%, and 70%. The shaping process is primarily influenced by the material removal rate, which increases as the duty cycle increases. However, it is important to note that a resistive barrier develops at the anode side, which restricts the material removal to a certain extent during electropolishing. The shaping of the Ni mould tools resulted in an increase in the fillet radius of up to 3.8 μm, a positive draft angle of 3.15°, and a width reduction of 11.4%, as observed during the optimization study.
- The polishing of Ni mould tools had a significant impact on the surface roughness (Sa) and surface shine (GU). The changes in surface roughness varied depending on the duty cycle employed. Specifically, for the 40% and 50% duty cycles, there was a reduction in surface roughness. However, for the 60% and 70% duty cycles, the surface roughness increased. Conversely, the surface shine showed an overall increase for all duty cycles. During the polishing process of the Ni mould tools, there was a noticeable reduction in surface roughness (Sa) for the 40% and 50% duty cycles, ranging from 5.5 to 8.6%. In contrast, for the 60% and 70% duty cycles, there was an increase in surface roughness from 42.8 to 51.3%. Additionally, the surface shine improved for all duty cycles, with an increase from 3 to 48.9%. These results highlight the effectiveness of the polishing process in enhancing the surface characteristics of the Ni mould tools.
- The validation of the optimized pulsed electropolished Ni mould tool was carried out through two key measurements: side wall roughness and replication of micro features on PMMA chips. Firstly, a PDMS casting of the Ni mould with linear micro channels was produced both before and after the electropolishing process. The examination of the linear side wall revealed a remarkable reduction in surface roughness of typically 16%. Secondly, hot embossing was employed to replicate the Ni mould with linear and star patterns onto PMMA chips. The quality of the resulting PMMA chips was significantly enhanced, as evidenced by the elimination of burrs, material pile up, and feature distortions observed during SEM analysis. These improvements are due to a reduction in friction and adhesion between the mould and polymer, contributing to defect-free replication of the micro features. The successful validation of the electropolished Ni mould tool through side wall roughness measurement and replication of micro features on PMMA chips further confirms its efficacy in improving the performance and efficiency of the micro injection moulding process.
